# Modulatory effects of *in ovo* delivery of galactooligosaccharide and *Lactiplantibacillus plantarum* on antioxidant capacity, gene expression, and selected plasma metabolite parameters of broiler chickens

**DOI:** 10.1007/s13353-024-00931-7

**Published:** 2024-12-12

**Authors:** Modou Mangan, Katarzyna Połtowicz, Cornelia C. Metges, Maria Siwek

**Affiliations:** 1 Department of Animal Biotechnology and Genetics, Bydgoszcz University of Science and Technology (PBS), Mazowiecka 28, 85-084 Bydgoszcz, Poland; 2 Department of Poultry Breeding, National Research Institute of Animal Production, Krakow-Ska 1, 32-083 Balice, Poland; 3 Research Institute for Farm Animal Biology (FBN), Nutritional Physiology, Wilhelm-Stahl-Allee 2, 18196 Dummerstorf, Germany

**Keywords:** Antioxidant, Broiler, Gene expression, Gut health, *In ovo*

## Abstract

A stable gut microbiota promotes a healthy gut and enhances immune function, antioxidant status, and metabolic activities in chickens. The present research work aimed to investigate the modulatory impacts of *in ovo* delivery of prebiotic and probiotic on oxidative stress, the intestinal transcriptome, and various plasma metabolites in chickens. Fertilized Ross 308 eggs were administered *in ovo* either with galactooligosaccharide (GOS) (3.5 mg/egg or *Lactiplantibacillus plantarum* (LP) 1 × 10^6^/egg on the 12th day of egg incubation. Three hundred viable Ross 308 broiler hatching eggs in total were randomly assigned to four groups, namely, the negative control not injected group, the group receiving physiological saline injections as the positive control, GOS, and LP. The analysis of genes associated with immune functions, antioxidants, barrier functions, and free fatty acid receptors were determined via qPCR. The analysis of the selected plasma blood metabolites was performed automatically with Pentra C 400. The antioxidant capacity of the chickens’ liver, breast muscle, and spleen was enhanced by the *in ovo* injection of GOS and LP. The immune-related gene expression levels were upregulated after *in ovo* stimulation with either GOS or LP which improved the gut health of broiler chickens. In addition, several genes related to gut barrier functions were upregulated, thus ensuring epithelial integrity. As for blood plasma metabolites, no adverse effects were observed. In summary, we report that *in ovo* stimulation with either GOS or LP stimulates the immune system and improves the antioxidant status and gut health of chickens with no negative impact on plasma blood metabolite indices.

## Introduction

Broiler chickens are among the most important sources of animal protein for humans, it is more affordable and is widely accepted in different regions and religions across the globe (Mottet and Tempio 2017). This has led to the intensification and commercialization of the broiler industry worldwide, with a rapid increase in production. However, this practice is associated with several consequences, such as metabolic disorders, pathogen infection, oxidative stress, morbidity, and mortality (Yang et al. 2024). In light of these adverse effects, leading to production losses in the broiler industry, the search for sustainable alternative strategies to maximize and ensure the continuous and efficient production of healthy and high-quality broiler chickens has gained much attention. In recent years, different probiotics and prebiotics have been developed and used via different strategies to promote poultry health and performance (Slawinska et al. 2020b; Wishna-Kadawarage et al. 2024). These bioactive substances are supplemented in the diet or water offered to the chickens or *in ovo* methods (Bednarczyk et al. 2016). However, the latter has received much attention due to early gut colonization and improvements in the immune system and health during embryonic development (Alagawany et al. 2018; Slawinska et al. 2020b; Das et al. 2021; Kpodo and Proszkowiec-Weglarz 2023).

Prebiotics, such as galactooligosaccharides (GOS), are food components that cannot be digested by the body’s own enzymes but have positive impacts on the host by directing the growth and activity of beneficial gut bacteria associated with improved gut health nutrient and absorption (Roberfroid 2007; Bertocchi et al. 2019; Slawinska et al. 2020b). Live microorganism known as probiotics improves the host’s health by enhancing the balance of the gut microbiota and improving overall health and welfare (Dunislawska et al. 2019). Among the *Lactobacillus species*, *Lactiplantibacillus plantarum* has been used as a probiotic in chicken feed and administered *in ovo* during embryonic development and has been reported to confer beneficial effects. *Lactiplantibacillus plantarum* is characterized by its ability to produce lactic acid and its resilience in the gastrointestinal tract (Jha et al. 2020; Fathima et al. 2022) and its antioxidant capacity in chickens (Yang et al. 2019). The use of 2,2-Diphenyl-1-picrylhydrazyl (DPPH) in vitro assay demonstrated that several probiotics such as *L. plantarum*, can scavenge free radicals and thereby counteract oxidative stress and subsequently alleviate multiple stressors, such as heat stress, pathogen infection, and metabolic disorders, later in the life of broiler chickens (Mu et al. 2018; Mounir et al. 2022; Yang et al. 2024).

In poultry, heat stress causes an increase in reactive oxygen species (ROS), leading to oxidative stress in chickens, ultimately causing an imbalance between oxidants and antioxidants (Wilson et al. 2014; Sumanu et al. 2023). while increasing the malondialdehyde levels (Zeng et al. 2014). It is reported that the supplementation of LP and GOS boosts the level of antioxidant expression in chickens (Slawinska et al. 2020b; Sumanu et al. 2023).

Although the *in ovo* injection of GOS or LP in chickens has already been tested (Slawinska et al. 2020b; Yang et al. 2024), results in the literature are inconsistent and the understanding of the associated biological and molecular processes is limited. Therefore the first aim of the present study was to evaluate the radical scavenging ability of the *Lactiplantibacillus plantarum* via in vitro studies. Secondly, we investigated the effects of GOS and LP administered *in ovo* on plasma biochemical indices and transcriptomic analysis of genes related to gut health, immune functions, and antioxidant activities in broiler chickens.

## Materials and methods

### Probiotic strains and culture conditions

The probiotics used in this research work (Table 1) were acquired from the JHJ Company (Nowa Wies, Gizalki, Poland). Before use, the probiotic cultures were kept at − 80 °C in 50% glycerol. All strains were cultured and maintained in MRS broth at 37 °C. A total of 6.82 g of MRS agar (de Man, Rogosa and Sharpe, Merck KGaA, Darmstadt) was dissolved in 100 mL of distilled water and stirred (IKA® RCT basic IKAMAG™ Safety Control Magnetic Stirrer) to dissolve the agar completely. Next, it was autoclaved at 121 °C for 15 min and vortexed for 10 s. Stock cultures of probiotic strains were established on agar plates, and the plates underwent a 24-h incubation period at 37 °C. A bacterial suspension was prepared for each strain in 10 mL of DeMan, Rogosa, and Sharpe broth (MRS) (Merck KGaA, Darmstadt). Subsequently, a 96-well microplate was filled with 250 µL of MRS broth and 10 µL of each bacterial suspension. The microplate was then incubated for 48 h at 37 °C under aerobic conditions. Three replicates of each sample were included for each of the three repetitions for each probiotic. MRS broth without inoculum was used as a control. Bacterial growth measurements (OD600) were performed every 12 h using a Multiskan™ FC Microplate Photometer and SkanIt software version 7.0 (Thermo Fisher Scientific, Waltham, MA). Microtiter plates were shaken for 10 s before the microplate readings were taken to ensure homogeneity in the samples.

**Table 1:** Concentrations of probiotics used for the 2,2-diphenyl-1-picrylhydrazyl (DPPH) DPPH test

Probiotic concentrations	
L. casei	*Lacticaseibacillus casei* 1.4 × 10^6^
	*Lacticaseibacillus casei* 7.0 × 10^5^
	*Lacticaseibacillus casei* 3.5 × 10^5^
L. plantarum	*Lactiplantibacillus plantarum* 4.4 × 10^6^
	*Lactiplantibacillus plantarum* 2.1 × 10^6^
	*Lactiplantibacillus plantarum* 1.0 × 10^6^
L. reuteri	*Limosilactobacillus reuteri* 7.9 × 10^6^
	*Limosilactobacillus reuteri* 3.9 × 10^6^
	*Limosilactobacillus reuteri* 1.9 × 10^6^
L. rhamnosus	*Lacticaseibacillus rhamnosus* 1.1 × 10^8^
	*Lacticaseibacillus rhamnosus* 5.5 × 10^7^
	*Lacticaseibacillus rhamnosus* 2.7 × 10^7^

### In vitro determination of the antioxidant activities of the selected probiotics

The list of *Lactobacillus* strains used in this study is provided in Table 1. To pre-select probiotics for *in ovo* injection, we used the 2,2-diphenyl-1-picrylhydrazyl (DPPH) (Sigma‒Aldrich, St. Louis, MO) assay to measure free radical scavenging activities according to (Kao and Chen 2006).

Briefly, 0.1 mM of DPPH was dissolved in 100 mL of ethanol. The mixture was vigorously shaken and left to react for 30 min at room temperature in the dark. It was always used fresh on the day of analysis. Prior to the DPPH assay, the *Lactobacillus* samples were serially diluted, and 10 µl of sample (with appropriate dilution), 190 µl of sample, and the control (200 µl of DPPH ethanolic solution) were added to a 96-well microtiter plate. The blank group contained MRS broth media and ethanol. The optical absorbance at 520 nm was measured in triplicate using a MultiskanTM FC Microplate Photometer. Using the following equation below, the percentage of free radical scavenging activity was determined.

$$\left(\%\right) \text {scavenging activity}= [(\text{Ac}-\text{As})/\text{Ac}] \times 100$$

where Ac is the absorbance of the control and As is the absorbance of the sample.

The results are expressed as the EC50 (μg/mL), which is the lowest antioxidant concentration needed to reduce 50% of the initial DPPH reaction from the moment the extract reached stability. Based on the growth curve and the DPPH assay results, the bioactive compound with the best growth and highest antioxidant activity was selected for *in ovo* application to validate its effects on Ross 308 broiler chickens. The prebiotic GOS was selected for *in ovo* application studies based on results of previous studies by our group showing its ability to mitigate heat stress in Ross 308 broilers (Slawinska et al. 2020b).

### Egg incubation and in ovo protocol

A total of three hundred (300) fertile Ross 308 broiler eggs were incubated under standard incubation conditions (Midi series I, Fest Incubators, Gostyń, Poland). On day 7 of embryonic development, eggs were taken out of the incubator and sterilized using 70% ethanol, then candled, and the infertile and dead embryos were discarded. The remaining fertile eggs were randomly allotted into four groups: negative control (NC), positive control (PC), GOS, and LP. Next, a 20G needle was used to make a hole in the air chamber of the eggs. Subsequently, *in ovo* injection was manually performed on the 12th day of egg incubation in all the groups except the NC group. A 0.2 mL sterile 0.9% physiological saline solution was injected into the PC group eggs while the GOS group eggs were injected with 3.5 mg of GOS/egg suspended in 0.2 mL of physiological saline and the LP group was injected with 10^6^ CFU of LP bacteria/egg suspended in 0.2 mL of physiological saline solution. After injection, each egg was sealed using organic glue (Elmer’s school glue, Elmer’s Products Inc., USA), and immediately returned to the incubator.

### Birds and housing

The experiment was conducted in compliance with the Ethics Committee for Experiments with Animals guidelines and the Polish Act on the Protection of Animals Used for Scientific or Educational Purposes regulations of January 15, 2015 (which was implemented by the European Parliament and Council of September 22, 2010, Directive 2010/63/EU on the protection of animals used for scientific purposes). All birds in each experimental group consisting of 32 birds/pens were housed in separate pens with similar optimized environmental conditions during the experiment. Water and feed were made available to the chickens at all times. The birds were fed the following three types of age-dependent diets throughout the experimental period: starter (1–21 days), grower (22–28 days), and finisher (29–35 days), consisting of 12.45, 13.01, and 13.01 MJ/kg of metabolizable energy and 22.3%, 20.2%, and 20.2% crude protein, respectively. The dietary mixtures were in accordance with broiler chicken dietary requirements (Smulikowska and Rutkowski, 2018). The initial environmental temperature in the pens was 32–33 °C on day one of life, and the temperature steadily reduced reaching approximately 21 °C at the end of the trial period.

### Sample collection

At the end of the rearing period, 8 birds per group (*n* = 24) with a final average body weight of 2.43–2.53 kg were randomly chosen. The birds were slaughtered by decapitation after being deprived of feed for 10 h and left to bleed for approximately 90 s. Following the slaughtering of each bird, two milliliters of blood were collected in K-EDTA tubes and centrifuged at 3000 × g for 15 min to extract plasma. Next, the plasma samples were immediately placed on dry ice and transported to the laboratory. Upon arrival, all the samples were kept at − 80 °C until analysis. In addition, cecal mucosa, liver, spleen, and breast muscle were collected and preserved in RNA stabilization reagent (fix RNA: E0280, EURx, Gdańsk, Poland) and transported at room temperature, and the fixed RNA was poured off and the tubes with the samples were kept at − 80 °C until use.

### RNA extraction, RT‒PCR, and qPCR gene expression analysis

Tissues were homogenized with a TissueRuptor homogenizer (990,890, Qiagen, Wrocław, Poland) and immersed in a tube containing 1 mL of RNA extracol solution (E3700, EURx, Gdańsk, Poland) for the RNA isolation procedure. Next, each sample was centrifuged using 0.2 mL of chloroform (112,344,305, Chempur, Piekary Śląskie, Poland). A commercial kit (Universal RNA purification kit (E3598, EURx, Gdańsk, Poland)) was used to carry out the subsequent steps of the RNA isolation process. A NanoDrop 2000 spectrophotometer (Thermo Scientific, Warsaw, Poland) was used to measure the quantity and quality of the RNA, while a 2% agarose gel was used to assess RNA integrity. RNA samples were stored at − 80 °C until use. Using the smART First Strand cDNA Synthesis Kit (0804, EURx, Poland), the RT-PCR process was performed following the manufacturer’s protocol. Next, the cDNA obtained was diluted to 100 ng/μl. Afterward, RT‒qPCR was carried out using a total volume of 10 μL. The reaction mixture included Maxima SYBR Green qPCR Master Mix (0401, EURx, Gdańsk, Poland), 1 μM of each primer, and 2 μl of diluted cDNA. Thermal cycling was conducted using a LightCycler II 480 (Roche Diagnostics, Basel, Switzerland). Each RT‒qPCR was carried out in two technical replicates in 96-well plates (4TI-0955, AZENTA, Genomed, Warszawa, Poland). The qPCR protocol for the gene expression analysis consisted of initial denaturation for 15 min (95 °C), followed by 40 cycles of amplification (denaturation at 95 °C for 15 s, annealing at 58 °C for 30 s, and elongation at 72 °C for 30 s).

The expression levels of the target genes were determined via geometric means of two housekeeping genes (*Actb* and *G6pdh*). The target genes analyzed for each tissue and the reference genes are listed in Table 2. The relative gene expression was calculated using the ΔΔCt method. The ΔCt of the control group was subtracted from the ΔCt of each of the treatment groups. The fold change (FC) of the target gene in the treatment group against the control group was calculated as 2^−∆∆Ct^.

**Table 2:** List of target genes used for qPCR gene expression analysis

Tissues	Gene	Primer Sequences (5′−3′)	References
Cecal mucosa	Claudin1 (*Cldn1*)	F: TCTTCATCATTGCAGGTCTGTC R: AACGGGTGTGAAAGGGTCAT	(Slawinska et al. 2019)
	Mucin 6 (*Muc6*)	F: TTCAACATTCAGTTCCGCCG R: TTGATGACACCGACACTCCT	(Slawinska et al. 2019)
	Avian beta defensin 1 (*Avbd1*)	F: AAACCATTGTCAGCCCTGTG R: TTCCTAGAGCCTGGGAGGAT	(Slawinska et al. 2019)
	Free fatty acid receptor 2 (*Ffar2*)	F: GCTCGACCCCTTCATCTTCT R: ACACATTGTGCCCCGAATTG	(Slawinska et al. 2019)
	Tight junction-associated protein 1 (*Tjap1*)	F: AGGAAGCGATGAATCCCTGTT R: TCACTCAGATGCCAGATCCAA	(Slawinska et al. 2019)
	Interleukin 1 beta (*Il1b*)	F: GGAGGTTTTTGAGCCCGTC TCGAAGATGTCGAAGGACTG	(Dunislawska et al. 2017)
	Interleukin 10 (*Il10*)	F: CATGCTGCTGGGCCTGAA R: CGTCTCCTTGATCTGCTTGATG	(Rothwell et al. 2004)
	Cathelicidin 2 (*Cathl2*)	F: AGGAGAATGGGGTCATCAGG R: GGATCTTTCTCAGGAAGCGG	(Slawinska et al. 2019)
Liver	Glutathione peroxidase- 1 (*Gpx1*)	F: TTGTAAACATCAGGGGCAAA R: ATGGGCCAAGATCTTTCTGTAA	(Akbarian et al. 2014)
	Heme oxygenase 1 (*Ho1*)	F: CTCAAGGGCATTCATTCG R: ACCCTGTCTATGCTCCTGTT	(Wu et al. 2019)
	Nuclear factor erythroid 2-related factor 2 (*Nrf*2)	F: ATCACCTCTTCTGCACCGAA R: GCTTTCTCCCGCTCTTTCTG	(Wu et al. 2019)
	Interleukin 1 beta (*Il1b*)	F: GGAGGTTTTTGAGCCCGTC TCGAAGATGTCGAAGGACTG	(Dunislawska et al. 2017)
	Occludin	F: TCATCCTGCTCTGCCTCATCT R: CATCCGCCACGTTCTTCAC	(Wu et al. 2019)
	Free fatty acid receptor 4 (*Ffar4*)	F: AGTGTCACTGGTGAGGAGATT R:ACAGCAACAGCATAGGTCAC	(Slawinska et al. 2019)
Breast muscle	Superoxide dismutase 1 (*Sod1*)	F: AGGGGGTCATCCACTTCC R: CCCATTTGTGTTGTCTCCAA	(El-Deep et al. 2014)
	Catalase (*Cat*)	F: GGGGAGCTGTTTACTGCAAG R: CTTCCATTGGCTATGGCATT	(El-Deep et al. 2014)
	Nuclear factor erythroid 2- related factor 2 (*Nrf2*)	F: ATCACCTCTTCTGCACCGAA R: GCTTTCTCCCGCTCTTTCTG	(Wu et al. 2019)
	Manganese superoxide dismutase (*Mnsod*)	F:TTCCTGACCTGCCTTACGACTAT R: CCAGCGCCTCTTTGTATTTCT	(Li et al. 2011)
	Zonula Occludens 1 (*Zo1*)	F:CTTCAGGTGTTTCTCTTCCTCCTC R:CTGTGG TTTCATGGCTGG ATC	(Chang et al. 2020)
Spleen	Cathelicidin 2 (*Cathl2*)	F: AGGAGAATGGGGTCATCAGG R: GGATCTTTCTCAGGAAGCGG	(Slawinska et al. 2019)
	Interleukin 4 (*Il4*)	F: GCTCTCAGTGCCGCTGATG R: GGAAACCTCTCCCTGGATGTC	(Sławinska et al. 2014a)
	Interleukin 8 (*Il8*)	F: CCACTGCTCCCTGGGTACAG R:TCAGAATTGAGCTGAGCC TTG	(Sławinska et al. 2014a)
	Interleukin 12p40 (*Il12p40*)	F: TTGCCGAAGAGCACCAGCCG R: CGGTGTGCTCCAGGTCTTGGG	(Brisbin et al. 2010)
	Reference genes		
	Actin, beta (*Actb*)	F: CACAGATCATGTTTGAGACCTT R: CATCACAATACCAGTGGTACG	(Sevane et al. 2014)
	Glucose-6-phosphate dehydrogenase (*G6pdh*)	F: CGGGAACCAAATGCACTTCGT R: GGCTGCCGTAGAGGTATGGGA	(Sevane et al. 2014)

### Blood plasma metabolite analysis

Blood plasma from eight 35 d old birds per each experimental group was randomly chosen to analyze metabolite concentrations and enzyme activities. An automatic enzyme analyzer (Pentra C 400, Axon Lab AG, Germany) was used to determine aspartate aminotransferase (AST): Kit No. A11A01629; alanine aminotransferase (ALT): A11A01627; high-density lipoprotein (HDL): A11A01636; low-density lipoprotein (LDL): A11A01638; total cholesterol: Kit No. A11A01634; triglyceride (TG): Kit No. A11A01640 (Horiba ABX), non-esterified fatty acid (NEFA): Kit No. 434–91,795 (Wako Chemicals GmbH, Neuss, Germany)); uric acid: Kit No. A11A01670; glucose: Kit No. A11A01667; lactose dehydrogenase (LDH): Kit No. A11A01871; and gamma-glutamyl transferase (GGT): Kit No. A11A01630 (Axon Lab AG, Reichenbach, Germany). These selected plasma metabolite parameters were analyzed at the Institute of Nutritional Physiology at the Research Institute for Farm Animal Biology (FBN), Dummerstorf, Germany.

### Statistical analysis

All the data were checked for distribution normality, presence of outliers, and homogeneity of variance using the Shapiro–Wilk test and Levene test, respectively. The DPPH in vitro results and plasma metabolites were analyzed using GraphPad Prism version 10.1.2. The one-way ANOVA was used for the DPPH in vitro data. To analyze the plasma metabolites, we used principal component analysis (PCA). Tukey’s HSD test was used to determine the differences between means (*P* < 0.05). The ΔCt values of every treatment group were compared with those of the control group for the gene expression analysis using GraphPad Prism and Student’s *t*-test to identify significant differences among the treatments (*P* < 0.05), and plotting of graphs was done with Microsoft Excel.

## Results and discussion

### DPPH antioxidant assay

**Fig. 1:**
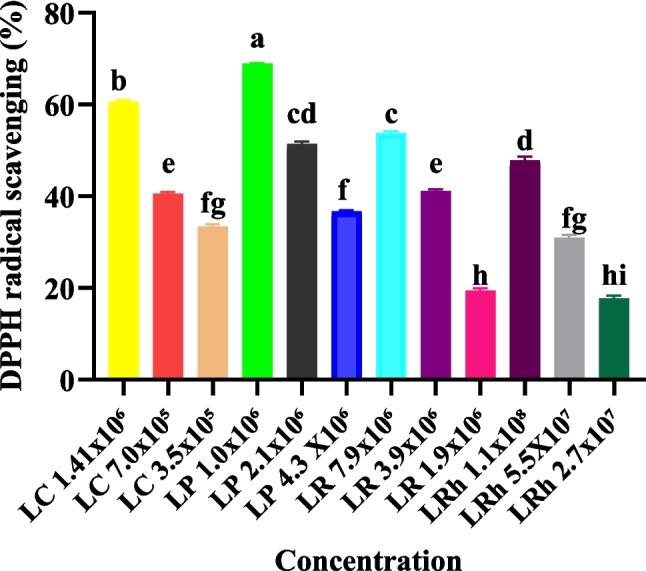
Free radical scavenging activities of the *Lactobacillus* species 2,2-diphenyl-1-picrylhydrazyl (DPPH). The results are expressed as means ± SEMs (*n* = 3). Different lowercase letters (a–i) indicate significantly different means (*P* < 0.05). LP, *Lactiplantibacillus plantarum*; LC, *Lacticaseibacillus casei*; LR, *Limosilactobacillus*; LRh, *Lacticaseibacillus* rhamnosus

The antioxidant potential and heat stress alleviation effects of prebiotic galactooligosaccharides were previously reported by our research group (Pietrzak et al. 2020). In this study, we tested the antioxidant capacity of several *Lactobacillus* species (Table 1) and found that *Lactiplantibacillus plantarum* 1.0 × 10^6^, exhibited the highest radical scavenging ability (68.89%) (*P* < 0.05) compared to the other probiotic bacteria (Fig. 1). Therefore we suggest that *Lactiplantibacillus plantarum* 1.0 × 10^6^ was able to influence the activities of *Sod*, *Cat*, *Nrf2*, and *Gpx1* and reduced heat stress in poultry (Mangan and Siwek 2023). In an in vitro study, it is reported that *Lactobacillus* species such as *L. plantarum* have antibacterial, antipathogen, and antifungal features (Li et al. 2012). In addition, *L. curvatus* and *L. plantarum* C88 demonstrated high antioxidant activities (59.67%) and (53.05%) respectively (Li et al. 2012; Zhang et al. 2017). (Mu et al. 2018) reported high antioxidant activities of *L. casei* Y3, Y4 and Y16 and *L. plantarum* Y41, Y42, and Y44 with the DPPH assay (*P* < 0.05).

### Gene expression analysis

#### Relative expression of different genes in cecal mucosal tissue

**Fig. 2:**
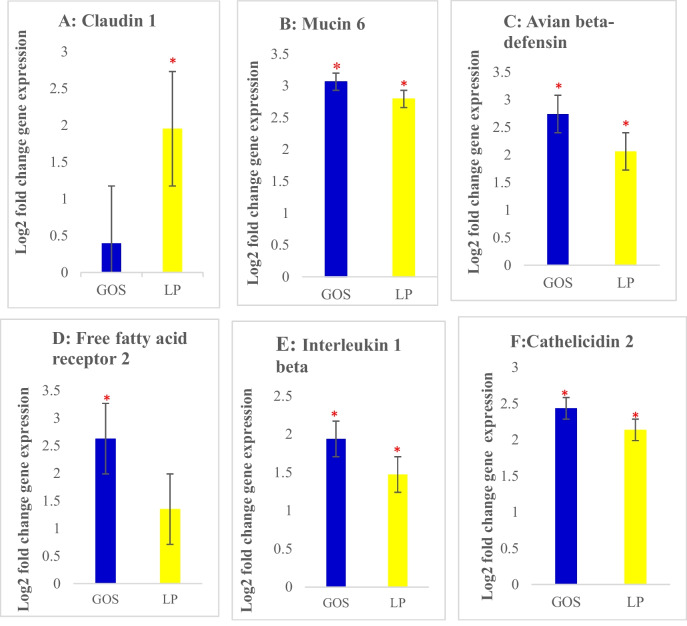
Gene expression levels in the cecal mucosa of chickens treated *in ovo* with either galactooligosaccharide (GOS) or *Lactiplantibacillus plantarum* (LP). **A** *Cldn1*, **B** *Muc6*, **C** *Avbd1*, **D** *Ffar2*, **E** *Il1b*, and **F** *Cathl2*. Error bars: ± SE. Red asterisks (*) indicate significant changes (*P* < 0.05)

In our study, we deduced that the *in ovo* administration of GOS led to significant upregulation (*P* < 0.05) of *Muc6*, *Avbd1*, *Ffar2*, *Il1b*, and *Cathl*2 (Fig. 2B, C, D, E and F ). On the other hand, *Cldn1*, *Avbd1*, *Il1b,* and *Cathl2* were upregulated (*P* < 0.05) by the *in ovo* administration of LP (Fig. 2A, B, C, E and F ). The *Muc6* gene was significantly upregulated (*P* < 0.05) in both of our treatment groups when compared to the positive control group. The *Muc6* gene is part of the mucus layer and is responsible for mucin secretion and plays an integral part in gut barrier functions (Forder et al. 2012); therefore, GOS and LP delivered *in ovo* were able to ensure the production of mucin in the cecal mucosa, thus conferring protection to chickens against pathogen infection. During the innate immune response, the *Avbd1* gene regulates the secretion of avian beta defensin1 which plays a crucial role in the inhibition of pathogens (Zhang and Sunkara 2014; Lyu et al. 2020). Even though high expression levels of *Avbd1* are prominent during infection, SCFAs like butyrate and acetate influence and promote defensin synthesis in epithelial cells without causing any inflammation or dysbiosis (Zhang and Sunkara 2014; Chen et al. 2020; Wishna-Kadawarage et al. 2024). According to our result, no form of inflammation was found in the cecal mucosa that might affect the chicken’s gut health. Therefore, the upregulation of the *Avbd1* gene may be caused by increased SCFA production through the modulation of the gut microbiota (Wishna-Kadawarage et al. 2024). In addition, our results revealed that *in ovo* delivery of GOS leads to the upregulation of *Ffar2*; therefore, it could be suggested that this gene plays a vital role in metabolic activities and immune cell recruitment in chicken cecal mucosa and subsequently modulates the gut microbiota (Slawinska et al. 2019). The *Ffar2* and *Ffar4* are nutrient-sensing genes that significantly influence the production of immune cells via SCFA production (Burns and Moniri 2010; Den Besten et al. 2013; Corrêa-Oliveira et al. 2016; Alvarez-Curto and Milligan 2016; Kolodziejski et al. 2018; Schlatterer et al. 2021). The *Cathl2* gene also supports gut barrier functions and regulates the inflammatory immune response (Volf et al. 2017; Slawinska et al. 2019). Our results confirmed that GOS and LP protect the gut barrier and reduce the risk of pathogen infection by increasing *Cathl2* expression (Fig. 2F). Interestingly, we found significant upregulation of *Il1b* in both GOS and LP (*P* < 0.05). *Il1b* plays a pivotal role in both proinflammatory cytokine production and protection against infection and therefore improves chicken gut health (Slawinska et al. 2019). According to (Khosravi and Mazmanian 2013; Slawinska et al. 2019), the gut of animals colonized by beneficial bacteria ensures a healthy gut which is correlated with high production of *IL1b*. *Cldn1* is a component of tight junctions that participates in preventing epithelial wall/cell permeability (Kawabe et al. 2001). In our study, LP enhanced *Cldn1* expression and therefore ensured epithelial cell integrity (Fig. 2A). Surprisingly, *in ovo* stimulation of either GOS or LP had no significant effect on *Tjap1* or *Il10*, suggesting that their functions were not compromised (Fathima et al. 2022).

#### Relative expression of different genes in splenic tissue

**Fig. 3:**
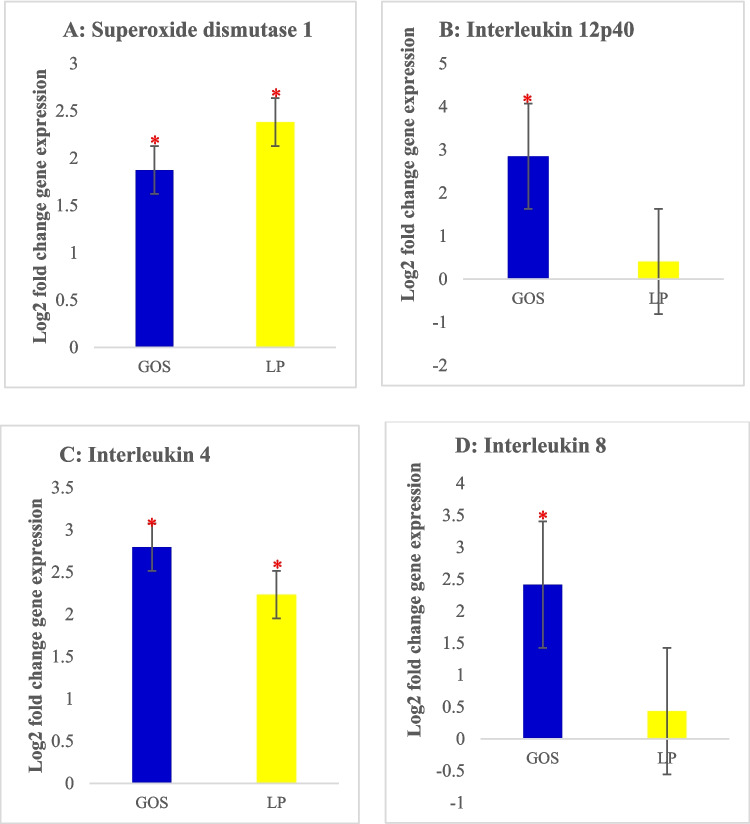
Gene expression levels in the splenic tissue of chickens treated *in ovo* with either galactooligosaccharide (GOS) or *Lactiplantibacillus plantarum* (LP). **A** *Sod1*, **B** *Il12p40*, **C** *Il4*, and **D** *Il8*. Error bars: ± SE. Red asterisks (*) indicate significant changes (*P* < 0.05)

With regard to the spleen, we observed a significantly increased (*P* < 0.05) expression levels of *Sod*, *Il12p40*, *Il4,* and *Il8* (Fig. 3A, B, C and D ) while the expression of *Il2* and *Cathl2* genes were not significantly affected.

*Il12p40* encodes the *p40* subunit, which is a key component of both *Il12* and *Il23*. In our study, the upregulation of *Il12p40* in GOS *in ovo-*treated chickens may be explained by the ability of GOS to produce high amounts of *Il12* and *Il23* by activating certain splenic antigen-presenting cells, namely dendritic cells and macrophages (Slawinska et al. 2020a). In the literature, *Lactobacillus salivarius* with GOS and *Lactiplantibacillus plantarum* with RFO delivered *in ovo* were shown to upregulate the immune-related genes, including *Il12p40* (Sławinska et al. 2014b; Dunislawska et al. 2017). A different study by (Dunislawska et al. 2019) reported that *in ovo* injection of a synbiotic consisting of *Lactobacillus salivarius* and GOS increased the gene expression levels in the spleens of chickens while *Lactiplantibacillus plantarum* with RFO had no effects on gene expression. The *Il4* gene was highly expressed (*P* < 0.05) in both of our treatment groups and this may be explained by the ability of GOS and LP to modulate the gut microbiome through IgA-mediated mechanisms and regulation of the peripheral immune system in the spleen (Sławinska et al. 2014a, b). Although the expression of *Il8* is associated with infection, the *Il8* gene is often involved in routine immune regulation and homeostasis and is important in recruiting immune cells such as heterophils to the spleen (Jarosinski et al. 2005; Yu et al. 2020; Pietrzak et al. 2020; Elnagar et al. 2021). The *Sod1* is the first line of defense against oxidative stress; thus it stabilizes the oxidant/antioxidant equilibrium by catalyzing the dismutation of superoxide radicals to hydrogen peroxide. We observed significant differences in the expression levels (*P* < 0.05) of *Sod1* in both of our treatment groups. Similarly, (Pietrzak et al. 2020; Ncho et al. 2021) reported an upregulation of *Sod* in heat-stressed birds. Therefore, we suggest that GOS and LP possess antioxidant potential and can alleviate oxidative stress in chickens.

#### Relative expression of different genes in breast muscle

**Fig. 4:**
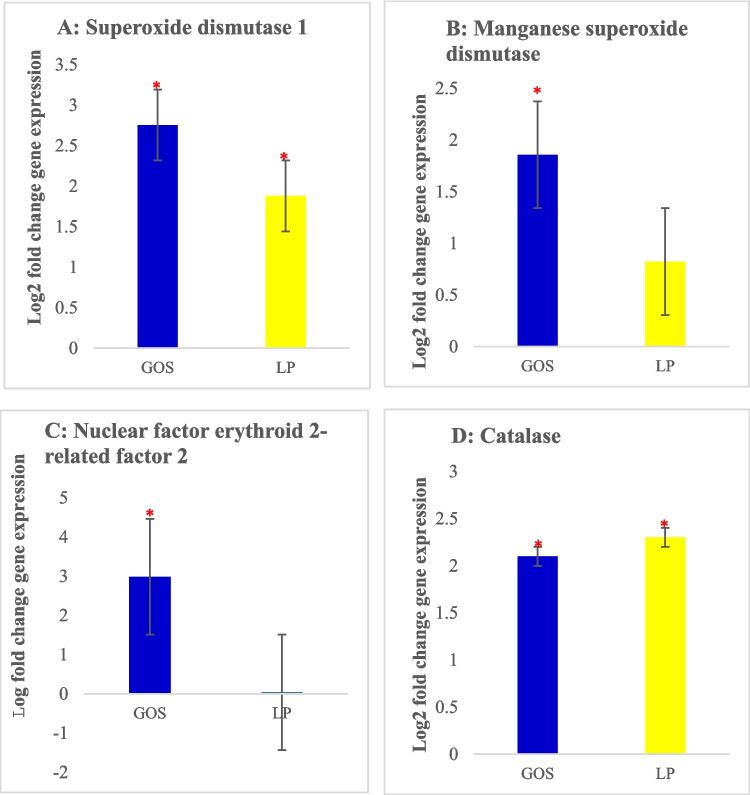
Gene expression levels in the breast muscle of chickens treated *in ovo* with either galactooligosaccharide (GOS) or *Lactiplantibacillus plantarum* (LP) **A** *Sod1*, **B** *Mnsod*, **C** *Nrf2*, and **D** *Cat*. Error bars: ± SE. Red asterisks (*) indicate significant changes (*P* < 0.05)

In our study, we demonstrated the *in ovo* delivery of GOS and LP increased the expression levels of *Gpx1*, *Cat*, *Sod*1, *Mnsod*, and *Nrf2* with no significant effect on Ho1 in chicken breast muscle. Oxidative stress destabilizes antioxidant levels in chickens, thereby causing an increase in *Mda* levels (Georgieva et al. 2006). Due to global warming and increasing temperatures worldwide strategies to alleviate oxidative stress and heat stress have gained much attention in the poultry industry (Mangan and Siwek 2023). Probiotic bacteria such as *Lactobacillus spp*. can activate the *Nrf2* pathway and other antioxidants such as catalase (*Cat*). Hydrogen peroxide is broken down into water and oxygen by the catalase antioxidant enzyme thereby preventing the accumulation of ROS (Surai et al. 2019; Karaca et al. 2022). The *in ovo* stimulation of GOS upregulated both *Cat* and *Nrf2*, (Surai et al. 2019). In our study, we found that GOS and LP significantly upregulated the antioxidants tested in chicken breast muscle suggesting that the oxidant/antioxidant balance of the chickens in this group was well-balanced while *Nrf2* was not affected (Fig 4). Similarly, several studies reported that *in ovo* delivery of prebiotics and probiotics upregulated the expression pattern of *Sod* and *Mnsod*, thereby reducing oxidative stress (Bai et al. 2017; Cheng et al. 2017; Cao et al. 2019; Pietrzak et al. 2020).

#### Relative expression of different genes in liver tissue

**Fig. 5:**
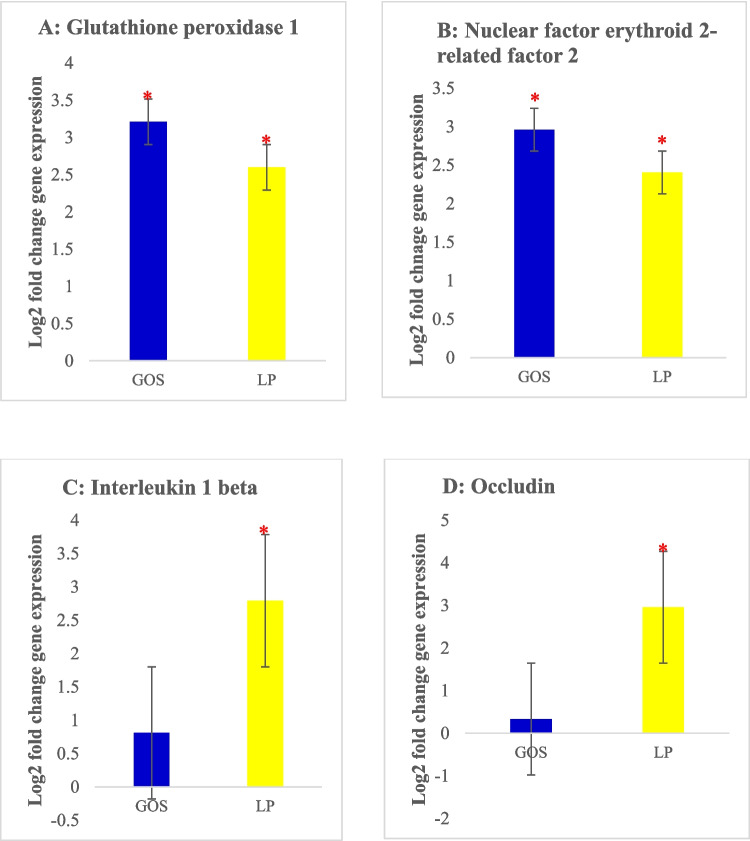
Gene expression levels in the liver of chickens treated *in ovo* with either GOS, galactooligosaccharide group; LP, *Lactiplantibacillus plantarum* group **A** *Gpx1*, **B** *Nrf2*, **C** *Il1b*, and **D** Occludin. Error bars: ± SE. The red asterisk (*) indicates significant changes (*P* < 0.05)

Our results showed that *in ovo* administration of GOS increased the expression levels (*P* < 0.05) of *Gpx1* and *Nrf2* (Fig. 5A and B ) in the livers of *in ovo*-treated chickens. Surprisingly, GOS affects *Nrf2*, occludin, *Ho1*, or *Ffar4* expression levels. On the other hand, LP led to the upregulation (*P* < 0.05) of *Gpx1*, *Nrf2*, *Il1b*, and occludin genes, while no significant effects were found on *Ho1* and *Ffar4* genes. The upregulation of the *Gpx1* gene by GOS and LP plays a vital role in the detoxification of hydrogen peroxide and lipid peroxides in poultry, thereby preventing oxidative stress. The positive effects exerted by GOS and LP may be explained by the modulatory ability of antioxidant enzymes and transcription factors involved in the *Nrf2* and *Gpx1* pathways (Surai et al. 2019; Gao et al. 2023).

*Il1b* and occludin were not highly expressed in GOS *in ovo*-treated chickens. However, *Il1b* and occludin were significantly upregulated (*P* < 0.05) upon *in ovo* supplementation with LP. Immune cells release the proinflammatory cytokine* Il1b* in response to infections or tissue damage*.* The high expression of *Il1b* suggests that *L. plantarum* triggered an inflammatory response in the liver, potentially due to its recognition as a foreign microorganism by the host’s immune system. However, this inflammatory response may be beneficial, as probiotics such as *L. plantarum* are known to induce a mild inflammatory response that can prime the immune system and enhance its ability to fight off harmful pathogens (Wang et al. 2018; Gao et al. 2022).

### Plasma blood metabolite analysis

**Fig. 6:**
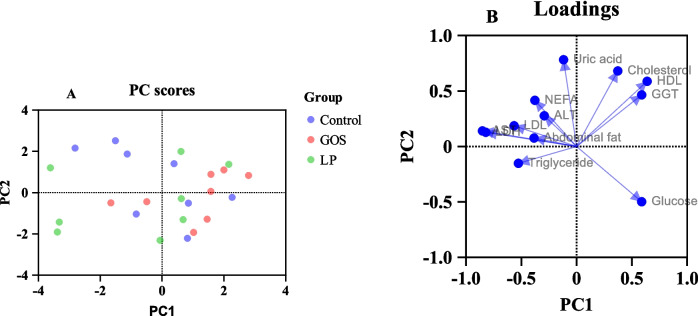
**A** Principal component analysis (PCA) plot of PCA score **B** and variables/plasma metabolite parameters. An individual refers to a sampled bird per treatment, while a variable is a biological parameter analyzed. The individuals have been colored according to treatment C, control; GOS, galactooligoaccharide; or LP, *Lactiplantibacillus plantarum*

The *in ovo* administration of either GOS or LP had no significant effects on the measured plasma metabolite parameters (Table 3). Surprisingly, our study, showed that the LP group had higher LDL than the PC and GOS groups. The high LDL concentration may be explained by the ability of LP to trigger compensatory mechanisms in lipid metabolism thus temporarily increasing lipid production. LDL cholesterol is mainly synthesized in the liver and plays an essential role in the transportation of lipids to peripheral tissues, however, when lipid metabolism is altered due to metabolic stress or other stressors, the liver may increase the production of LDL to ensure lipid homeostasis and therefore avert the accumulation of excess lipids in other tissues (Trapani et al. 2012). Additionally, our result showed an increased LDL which correlates to a numerically increased AST in the LP group compared to the GOS and PC groups (Table 3). In chickens, an increased level of AST could indicate mild liver stress. The slightly higher AST in the LP group may potentially be associated with increased lipid metabolism and LDL cholesterol (Lee et al. 2022). However, according to our Principal Component Analysis (PCA) result, the increased levels of LDL and AST in the LP group did not cause any negative effects on the chickens. The (PCA) was performed to study the effects of *in ovo* stimulation of GOS and LP on chicken plasma metabolites and enzymes. According to our results, no clear separation was observed between samples from the three groups (samples dot plot; Fig. 6A). The variable arrow plot (Fig. 6B) did not show a clear separation between the experimental groups for the various parameters. However, all the studied parameters tend to conglomerate together and were positively correlated, except for cholesterol, HDL, GGT and glucose. Based on the results, there were no substantial differences in metabolites between the groups. When chickens are exposed to stressful conditions such as oxidative stress, infections, and lipolysis, several metabolic changes may occur and consequently increase NEFA levels (Abramowicz et al. 2019). Our PCA revealed no significant effects on NEFA levels despite the ANOVA results showing that the control group had a numerically greater NEFA concentration than the GOS and LP groups. The unchanged NEFA levels observed in our study could be regarded as a positive effect, according to (Verago et al. 2001), lower/unchanged NEFA levels may indicate the ability of chickens to adapt to their environment or other stressors. The other parameters remain unaffected indicating that the *in ovo* stimulation of GOS or LP did not cause any negative effect on the health of the chickens (Table 3) and (Fig. 6A) which was confirmed by the transcriptomic analysis and our production data results (body weight, FCR, and feed intake M. Mangan et al., personal communication).

**Table 3:** Effect of *in ovo* administration of GOS and LP on chicken plasma metabolites

Parameters	Treatments			*P*-value
	Control	GOS	LP	
ALT (U/L)	10.38 ± 3.25	10.13 ± 1.73	10.75 ± 1.67	0.865
AST (U/L)	601.64 ± 248.55	452.46 ± 146.77	677.41 ± 278.62	0.167
HDL (mmol/L)	2.11 ± 0.19	2.18 ± 0.32	2.06 ± 0.36	0.708
LDL (mmol/L)	0.48 ± 0.13	0.41 ± 0.08	0.62 ± 0.20	0.022
Cholesterol (mmol/L	3.31 ± 0.25	3.34 ± 0.33	3.40 ± 0.45	0.884
Glucose (mmol/L	15.75 ± 4.03	18.00 ± 4.50	14.75 ± 4.20	0.307
GGT (U/L)	11.98 ± 1.48	12.77 ± 0.63	12.18 ± 0.81	0.313
LDH (U/L)	2300.13 ± 1583.05	1473.61 ± 948.33	2614.04 ± 2026.84	0.176
NEFA (µmol/L)	882.13 ± 362.18	676.00 ± 147.77	532.75 ± 124.26	0.055
TG (mmol/L)	0.46 ± 0.08	0.47 ± 0.18	0.59 ± 0.16	0.187
UA (µmol/L)	304.38 ± 101.41	241.13 ± 98.73	203.13 ± 99.78	0.148

## Conclusion

The present study provided evidence suggesting that the *in ovo* administration of either GOS or LP may positively influence gut health and immune functions in broiler chickens as indicated by changes in mRNA expression of relevant genes. Additionally, GOS and LP delivered *in ovo* prevented oxidative stress as indicated by the upregulation of certain antioxidants, such as *Cat*, *Sod1*, *Mnsod*, and *Nrf2*. Furthermore, the *in ovo* administration of either GOS or LP did not cause any negative effects on the selected plasma metabolites, indicating that the chickens were healthy and in good condition. Based on these findings, we showed that the *in ovo* administration of GOS and LP on the 12th day of egg incubation may provide long-lasting beneficial effects on chicken gut health, immunity, and antioxidant status. Overall, our results suggest that the *in ovo* administration of GOS (3.5 mg/egg) significantly influenced the gut health, immune functions, antioxidant activity and performance of chickens more than the *in ovo* injection of 1 × 10^6^ *Lactiplantibacillus plantarum*. We suggest that further research be conducted to understand the mechanisms involved in the antioxidant, molecular, and biological processes in specific chicken tissues and their effects on chicken health. Additionally, while our results showed beneficial effects of *in ovo* administration of GOS and LP as demonstrated by the mRNA expression levels of the selected genes, we suggest that further studies on enzymatic activities and protein expression levels be performed in other to understand their effects on chicken health and performance.

## Data Availability

All the data used in this study are available from the corresponding author upon reasonable request.

## Abbreviations

AC: Absorbance of control
*Actb*: Actin, beta
ALT: Alanine transaminase
ANOVA: Analysis of variance
AS: Absorbance of sample
AST: Aspartate aminotransferase
*Avbd1*: Avian beta defensin 1
*Cat*: Catalase
*Cathl2*: Cathelicidin 2
CFU: Colony forming units
cDNA: Complementary DNA
*Cldn1*: Claudin 1
DNA: Deoxyribonucleic acid
DPPH: 2,2-Diphenyl-1-picrylhydrazyl
EU: European Union
ED: Embryonic development
EC50: Half maximal effective concentration
*Ffar2*: Free fatty acid receptor 2
*Ffar4*: Free fatty acid receptor 4
*G6pdh*: Glucose-6-phosphate dehydrogenase
GGT: Gamma-glutamyl transferase
GOS: Galactooligosaccharide
*Gpx*1: Glutathione peroxidase 1
HDL: High-density lipoprotein
*Ho1*: Heme oxygenase 1
*Il1b*: Interleukin 1 beta
*Il4*: Interleukin 4
*Il8*: Interleukin 8
*Il10*: Interleukin 10
*Il12p40*: Interleukin 12p40
LAB: Lactic acid bacteria
LC: *Lacticaseibacillus* *casei*
LDH: Lactose dehydrogenase
LDL: Low-density lipoprotein
LP: *Lactiplantibacillus* *plantarum*
LR: *Limosilactobacillus* *reuteri*
LRh: *Lacticaseibacillus* *rhamnosus*
mg: Milligram
MJ/Kg: Mega Jules per kilogram
mL: Milliliter
mM: Millimolar
*Mnsod*: Manganese superoxide dismutase
MRS agar: De Man-Rogosa-Sharpe agar
*Muc6*: Mucin 6
NC: Negative control
NEFA: Non-esterified fatty acid
nm: Nanometer
*Nrf*2: Nuclear factor erythroid 2-related factor 2
OD600: Optical density at 600 nm
PC: Positive control
PCA: Principal component analysis
PCR: Polymerase chain reaction
qPCR: Quantitative polymerase chain reaction
RNA: Ribonucleic acid
rpm: Revolutions per minute
SCFA: Short-chain fatty acids
SD: Standard deviation
SE: Standard error
SEM: Standard error of means
*Sod1*: Superoxide dismutase 1
TG: Triglyceride
*Tjap1*: Tight junction-associated protein 1
UA: Uric acid
*Zo1*: Zonula occludens 1
